# Detection of Antibodies against Human and Plant Aquaporins in Patients with Multiple Sclerosis

**DOI:** 10.1155/2015/905208

**Published:** 2015-07-26

**Authors:** Aristo Vojdani, Partha Sarathi Mukherjee, Joshua Berookhim, Datis Kharrazian

**Affiliations:** ^1^Immunosciences Lab., Inc., 822 S. Robertson Boulevard, Suite 312, Los Angeles, CA 90035, USA; ^2^Department of Preventive Medicine, Loma Linda University, 24785 Stewart Street, Loma Linda, CA 92354, USA; ^3^Department of Mathematics, Boise State University, 1910 University Drive, Boise, ID 83725, USA; ^4^Department of Clinical Sciences, Bastyr University California, 4106 Sorrento Valley Boulevard, San Diego, CA 92121, USA

## Abstract

Multiple sclerosis (MS) is an autoimmune disease that affects the body's central nervous system. Around 90% of MS sufferers are diagnosed with relapsing-remitting MS (RRMS). We used ELISA to measure IgG, IgA, and IgM antibodies against linear epitopes of human and plant aquaporins (AQP4) as well as neural antigens in RRMS patients and controls to determine whether patients suffering from RRMS have simultaneous elevations in antibodies against these peptides and antigens. In comparison to controls, significant elevations in isotype-specific antibodies against human and plant AQP4 and neural antigens such as MBP, MOG, and S100B were detected in RRMS patients, indicating a high correlation in antibody reaction between plant aquaporins and brain antigens. This correlation between the reactivities of RRMS patients with various tested antigens was the most significant for the IgM isotype. We conclude that a subclass of patients with RRMS reacts to both plant and human AQP4 peptides. This immune reaction against different plant aquaporins may help in the development of dietary modifications for patients with MS and other neuroimmune disorders.

## 1. Introduction

Multiple sclerosis (MS) is characterized by the demyelination of a nerve's protective myelin sheaths in the brain and spinal cord, which occurs due to inflammation and attack by the body's own immune system [1, 2]. This myelin damage disrupts the communication between the brain and the rest of the body. Symptoms may include fatigue, vertigo, cognitive impairment, focal cortical deficits, unilateral painful loss of vision, postural and action tremor, dysarthria, limb incoordination and gait ataxia, diplopia, oscillopsia, pseudobulbar palsy, and bladder dysfunction. In 1996, the United States National Multiple Sclerosis Society described 4 clinical courses of the disease [3]. In 2013, this set of courses was reviewed by an international panel [4], resulting in the recognition of 4 main phenotypes of MS. The first type, relapsing-remitting multiple sclerosis (RRMS), affects around 90% of people who have MS. The defining elements of RRMS are episodes of acute worsening of neurologic function followed by a variable degree of recovery, with a stable course between attacks [3]. The remaining 10% have one of these three progressive forms: secondary progressive (SPMS), primary progressive (PPMS), and progressive relapsing (PRMS).

Aquaporin 4 (AQP4) is a class of water channels found in many cells of the body including the stomach, brain, lung, and skeletal muscle [5]. AQP4 is the predominant water channel in the central nervous system and is expressed in ependymocytes, endothelial cells, and astrocyte foot processes at the blood-brain barriers (BBB), but not in neurons [6, 7]. In the brain, AQP4 is believed to have a role in maintaining homeostasis and water exchange, electrical activity, and modulation of neuronal transmission and excitability [8, 9].

Neuromyelitis optica (NMO), or Devic's disease, is a severe inflammatory demyelinating disorder that affects the white and gray matter in the brain and is classically restricted to the optic nerves and spinal cord [10–12]. Studies have shown that a majority of patients with NMO produce antibodies against the extracellular domain of human AQP4 [13–17]. NMO meets all the formal criteria for an autoimmune etiology [18].

Although MS and NMO are now recognized as two distinct illnesses [18, 19] for years similar clinical manifestations led to one being misdiagnosed as the other or led some to think that NMO was a severe form of MS. The introduction of the NMO antibody permitted clearer differentiation between the two disorders and increased the accuracy of diagnosis [19].

In NMO lesions, products of complement cascade are found within astrocytes and macrophages [20]. Furthermore, using the immunofluorescence method and human AQP4 transfected cell lines, a disease-specific antibody against extracellular domains of human AQP4 designated as NMO-IgG has been detected in the blood of patients [13, 21–23]. The binding of IgG_1_ to human AQP4, in conjunction with complement activation, leads to a loss of human AQP4 functionality in lesions through complement-dependent cytotoxicity, tissue damage, and demyelination of the spinal cord and optic nerve, followed by opening of the BBB [11]. Since IgG_1_ against human AQP4 is produced in the blood, its access to the extracellular space of the CNS is greater when the BBB is compromised, which allows the antibodies to reach their target tissue [24]. This can lead to many complications, ranging from mild sensory disturbances to complete transverse myelitis with tetraplegia or paraplegia, sensory impairments, bladder-bowel dysfunction, and more [11, 24].

A variety of plant cells contain aquaporins, through which water can flow more rapidly inside the cells than by diffusing through the phospholipid bilayers [25]. In fact, 5 plant aquaporin families have been structurally and functionally well-studied and characterized [26, 27]. A recent study showed a significant similarity between the amino acid sequences of soy, spinach, corn, tomato, and tobacco with human aquaporin epitope 207–232 [28]. Furthermore, using ELISA, the researchers found that, in comparison to non-NMO samples, the NMO IgG serum reacted to both human and corn aquaporin peptides. However, that study was conducted by measuring only IgG in serum collected from 8 confirmed NMO patients, 1 probable NMO patient, and 9 non-NMO controls. Previous studies, including our own, have demonstrated that IgM and IgA antibodies have been detected against myelin basic protein (MBP), myelin oligodendrocyte glycoprotein (MOG), and other neural antigens in subgroups of patients suffering from MS and other neurologic disorders [29–34]. In fact, in a study on the importance of antibodies against myelin antigens in demyelination, Egg et al. showed that while IgG antibodies against MOG were 35%, IgM antibodies against MOG were the highest at 55%, while IgA had a respectable level at 21% [29]. Given the overlapping symptomatologies between NMO and MS, in this present study we extended the investigation to IgG, IgM, and IgA isotype antibody reactivity against 4 different plant sequences using 47 patients with RRMS. By measuring antibodies against MBP, MOG, and S100B along with human and plant aquaporins, we wanted to examine the association between the elevation in antibodies against plant aquaporins and neural antigens in patients with RRMS [29–34]. We hypothesized that, due to exposure to environmental proteins, antibodies to the linear epitopes of AQP4 peptides from humans and plants are detected in patients with RRMS. These findings warrant further investigation into the role of the environment in RRMS.

## 2. Material and Methods

### 2.1. Controls and MS Patients' Sera

Based on MRI scans, which show focal or confluent abnormalities in the brain's white matter, and clinical examinations that show a pattern of attack, complete or partial remission, and then a relapse at a future date, patients were classified as having RRMS and ranged from 22 to 63 years of age (male : female, 1 : 1). We chose only sera taken from patients upon their diagnosis or not more than 12 months after the initial diagnosis. These samples were purchased from Sanguine BioSciences, Inc. (Valencia, CA, USA) and BioServe (Beltsville, MD, USA). For comparison, 47 serum samples with matching age and sex from healthy donors were purchased from Innovative Research Inc. (Southfield, MI, USA). These individuals were qualified to donate blood based on a health questionnaire provided by the Food and Drug Administration (FDA). Each individual at the time of blood draw also did not exhibit any health complaints. Each blood sample was tested according to FDA guidelines for the detection of hepatitis B surface antigen, antibodies to HIV, antibodies to hepatitis C, HIV-1 RNA, hepatitis C RNA, and syphilis. None of the samples were positive for these antibodies or viral RNA.

### 2.2. Antigens and Peptides

MBP was purchased from Sigma Aldrich (St. Louis, MO); S100B was obtained from EMD Biosciences (San Diego, CA); human and plant aquaporin peptides, MOG peptide 21–40 with a purity of greater than 90%, and ovalbumin peptide 323–339 were ordered from Bio-Synthesis Inc. (Lewisville, TX). Monoclonal antibodies made against various aquaporin peptides were purchased from Sigma Aldrich (St. Louis, MO).

### 2.3. Detection of IgG, IgM, and IgA Antibodies by Enzyme Linked Immunosorbent Assay

MBP, S100B protein, MOG, and aquaporin peptides at a concentration of 1.0 mg/mL were each diluted 1 : 100 in 0.1 M carbonate-bicarbonate buffer, pH 9.5; 100 *μ*L was added to each well of a polystyrene flat-bottom ELISA plate. Plates were incubated overnight at 4°C and then washed three times with 300 *μ*L phosphate-buffered saline (PBS) containing 0.05% Tween 20, pH 7.4. The nonspecific binding of immunoglobulins was prevented by adding 2% BSA in PBS and incubated overnight at 4°C. Plates were washed as described above, and then serum samples from controls and RRMS patients were diluted 1 : 100 in 0.1 M PBS Tween containing 2% BSA, then added to duplicate wells, and incubated for 1 hour at room temperature. Plates were washed, and then alkaline phosphatase goat anti-human IgG, IgM, or IgA antibody (Jackson ImmunoResearch Laboratories, Inc. (West Grove, PA)) at an optimal dilution of 1 : 200 IgA, 1 : 500 IgG, and IgM in 2% BSA-PBS was added to each well; plates were incubated for an additional 1 hour at room temperature. After washing six times with PBS-Tween buffer, the enzyme reaction was started by adding 100 *μ*L of phosphatase substrate in 0.1 mL of diethanolamine buffer of 1 mg/mL containing 1 mM MgCl_2_ and sodium azide, pH 9.8. The reaction was stopped 45 minutes later with 60 *μ*L of 2N NaOH. The optical density (OD) was read at 405 nm by means of a microplate reader. Several control wells containing human serum albumin or ovalbumin peptide 323–339 were used for detection of nonspecific binding.

### 2.4. Determination of Specificity of Antibody Assay

For the determination of the specificity of the AQP4 antibody reaction, serial dilutions of sera as well as inhibition studies were conducted using specific and nonspecific antigens.

Different sera with high levels of IgG, IgM, or IgA antibodies against each aquaporin were diluted serially from 1 : 100 to 1 : 3200 and then applied to ELISA plates coated with the same peptide. After completion of the ELISA procedure, the recorded ODs were used for the generation of curves.

For inhibition, 5 different sera with a very high titer of IgG, IgA, or IgM antibody against human AQP4 were used in the inhibition study. In different test tubes, 1 mL of 1 : 100 diluted sera sample was preincubated with 100 *μ*L of diluent containing either 100 *μ*g HSA or human AQP4 or spinach, tomato, soy, or corn aquaporins. After mixing, the tubes were kept for 1 hour at 37°C water bath followed by 4-hour incubation at 4°C and then centrifuged at 3000 g for 10 mins. The supernatant was used for measuring IgG, IgA, or IgM antibody level against human AQP4, before and after absorption with different aquaporins.

### 2.5. Coefficients of Intra- and Interassay Variation

Coefficients of intra-assay variation were calculated by running five samples eight times within a single assay. Coefficients of interassay variation were determined by measuring the same samples in six consecutive assays. This replicate testing established the validity of the ELISA, determined the appropriate dilution with minimal background, and detected serum IgG, IgM, and IgA against different aquaporins. Coefficients of intra- and interassay variations for IgG, IgM, and IgA against all tested aquaporins were less than 15%.

### 2.6. Reaction of Antibody against AQP4 Peptides with Various AQP4

For measuring anti-AQP4 reactivity with different AQP4 peptides, we used ELISA similar to IgG, IgM, and IgA detection. Aside from the fact that mouse serum was used instead of human serum and the secondary antibody was enzyme-labeled anti-mouse IgG, all the other steps were the same.

### 2.7. Statistical Analysis

We first calculated Pearson's correlation coefficient between each isotype (lgG, lgA,. and lgM) of the food proteins (soy aquaporin, corn aquaporin, tomato aquaporin, and spinach aquaporin) and similar isotype of brain protein (MBP, MOG, S100B, and human aquaporin) in RRMS patients. Next, we performed simple regression analysis between each of those combinations and calculated their *p* values. If a *p* value is less than 0.05, we conclude that particular isotype of food protein significantly elevates similar isotype of that particular brain protein. Finally, we performed a two-way cluster analysis of Pearson's correlation coefficients between the antibody against food peptides and the brain proteins in RRMS patients. We performed all statistical analyses in the statistical software “R” (http://www.r-project.org/).

## 3. Results

### 3.1. Detection of Antibodies

**Figure 1:**
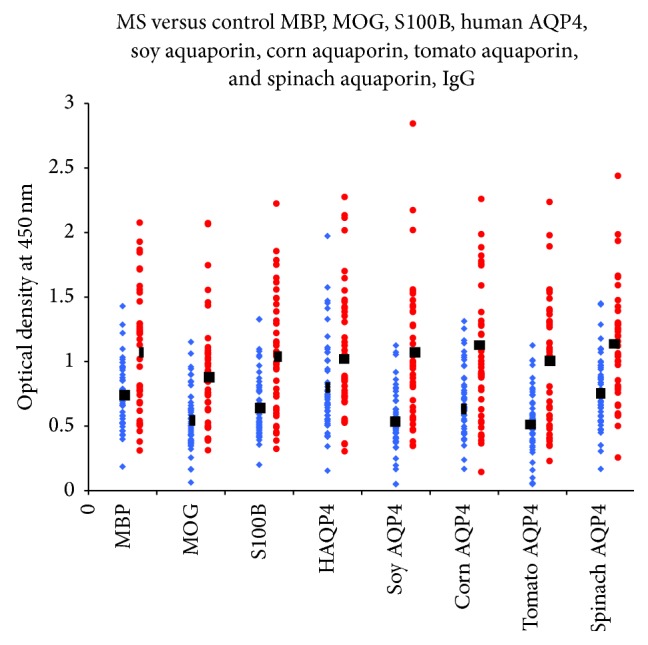
IgG antibody values of RRMS patients (blue diamond) versus controls (red circle): MBP, MOG, S100B, human AQP4, soy aquaporin, corn aquaporin, tomato aquaporin, and spinach aquaporin. The levels of serum IgG antibodies against almost all tested antigens were significantly higher in patients than in controls.

**Figure 2:**
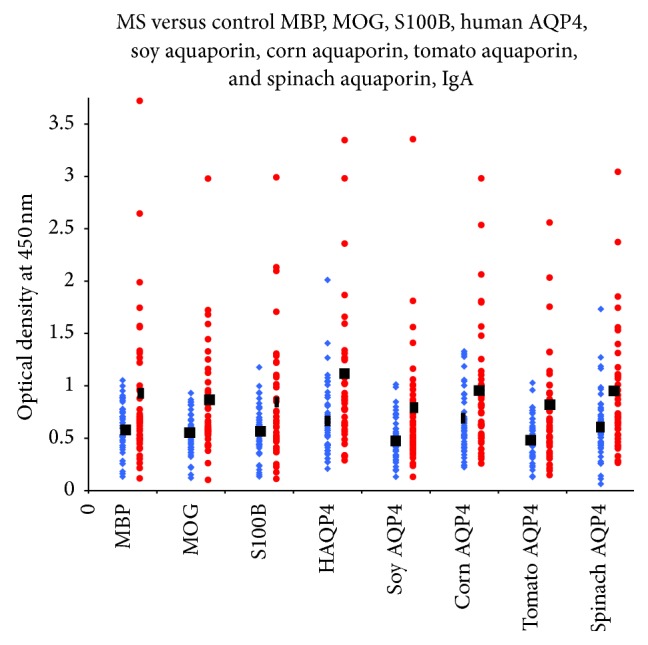
IgA antibody values of RRMS patients (blue diamond) versus controls (red circle): MBP, MOG, S100B, human AQP4, soy aquaporin, corn aquaporin, tomato aquaporin, and spinach aquaporin. The levels of serum IgA antibodies against all tested antigens were significantly higher in patients than in controls.

**Figure 3:**
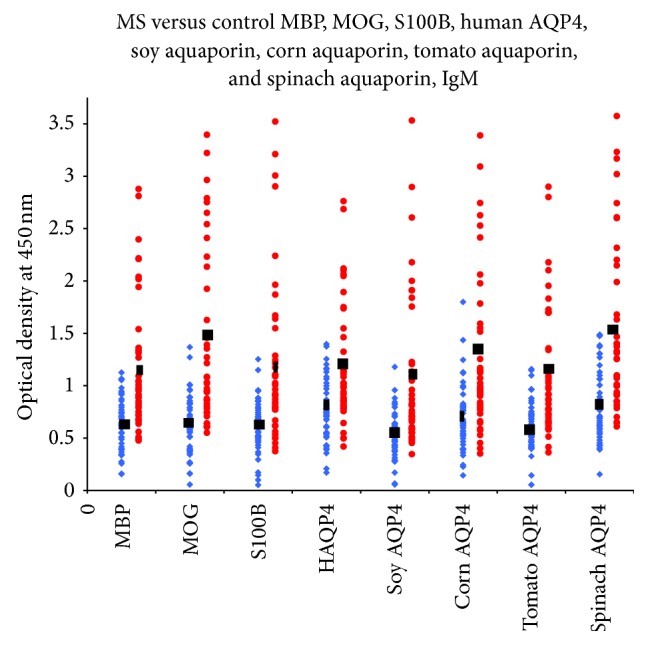
IgM antibody values of RRMS patients (blue diamond) versus controls (red circle): MBP, MOG, S100B, human AQP4, soy aquaporin, corn aquaporin, tomato aquaporin, and spinach aquaporin. The levels of serum IgM antibodies against all tested antigens showed even more significant differences between the control and patient groups, with all eight antigens showing *p* < 0.0001.

Sera from 47 patients with RRMS and 47 healthy controls were evaluated by ELISA to measure IgG, IgA, and IgM antibodies against both plant and human aquaporins, MBP, MOG, and S100B. Results presented as low and high OD values with the mean ± standard deviation (SD) are summarized in Table 1. The ODs for IgG antibody values obtained with 1 : 100 dilution of healthy control sera ranged from 0.05 to 1.97, varying according to subjects and antigens (Figures 1–3). The mean ± SD of these values ranged from 0.52 ± 0.26 to 0.81 ± 0.36. The corresponding IgG OD values for the sera from RRMS patients ranged from 0.15 to 2.84, while the mean ± SD ranged from 0.91 ± 0.45 to 1.15 ± 0.48 (Table 1). For the levels of IgG antibody against almost all the eight tested antigens, the differences in mean ± SD in patients versus controls were highly significant (*p* < 0.0001) (Figure 1). The results for IgA antibodies against neural antigens and various aquaporins in the sera of controls and RRMS patients are also shown in Table 1, as well as in Figure 2. The levels of serum IgA antibodies against all tested antigens were also significantly higher in patients than in controls. The mean ± SD for controls ranged from 0.49 ± 0.20 to 0.67 ± 0.34. The mean ± SD for patients ranged from 0.76 ± 0.54 to 1.11 ± 0.68. The levels of IgM antibody against the neural antigens and plant aquaporins were also examined in both groups. The individual test results shown in Figure 3, as well as the mean ± SD depicted in Table 1, showed even more significant differences between the control and patient groups, with all eight antigens showing *p* < 0.0001. We examined the data based on male and female subjects and also found a significant difference between male patients versus male controls and female patients versus female controls (*p* < 0.0001). However, no significant difference was detected between male and female patients (*p* > 0.05).

### 3.2. Statistical Analysis of the Data for Investigating Association between the Food Proteins and the Brain Proteins in RRMS Patients

**Figure 4:**
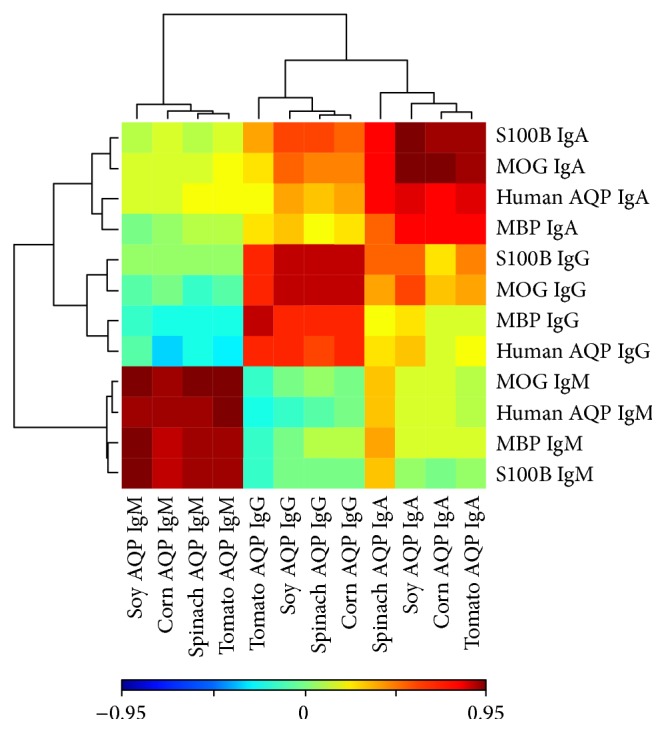
IgA, IgM, and IgG isotypes are clustered together with high correlations among the aquaporin peptides and brain proteins in each isotype.

We tested whether there were significant associations between the elevations of each antibody isotype (IgG, IgA, and IgM) of the brain proteins (MBP, MOG, S100B, and human aquaporin) with the corresponding antibody isotype of the food proteins (soy, corn, tomato, and spinach aquaporins) in RRMS patients. We fitted simple linear regression models between each such pair, and calculated the *R* ^2^ values and the *p* values. The summary of the results is presented in Tables 2, 3 and 4. From the tables we see that all of those food proteins significantly elevate similar isotypes of those four brain proteins or peptides in RRMS patients.  Figure 4 presents the result of a two-way cluster analysis of Pearson's correlation coefficients between the food and brain proteins or peptides where we see that IgG, IgA, and IgM isotypes are clustered together with high correlations among the food and brain proteins or peptides in each isotype. While there was a correlation between the reactivities of the MS patients' sera to neural cell antigens and plant aquaporins, the differences in IgM antibody reactivity between the two groups was the most significant (Figure 4).

### 3.3. Specificity of Antibodies

**Figure 5:**
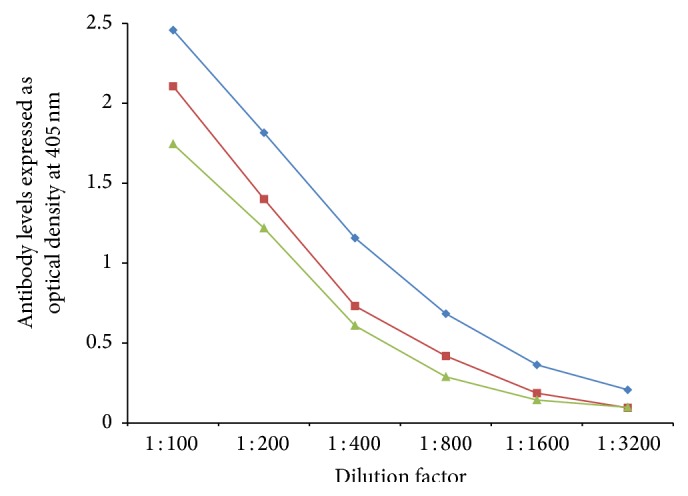
Serial dilution of IgG (blue diamond), IgA (green triangle), and IgM (red square) antibody against AQP4.

**Figure 6 fig6:**
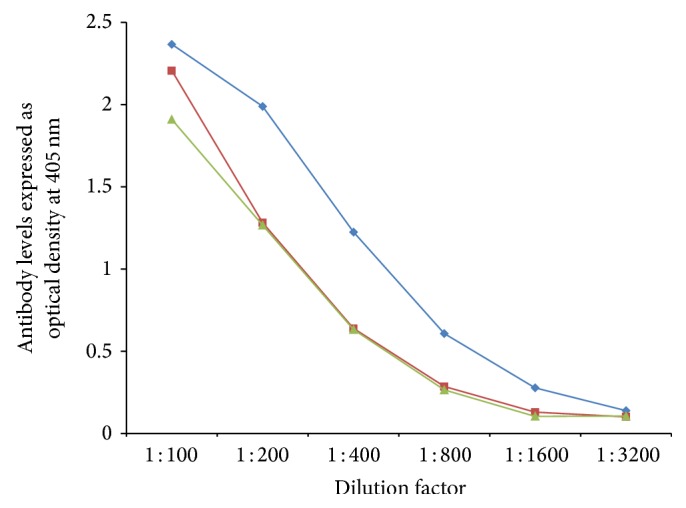
Serial dilution of IgG (blue diamond), IgA (green triangle), and IgM (red square) antibody against tomato.

**Figure 7 fig7:**
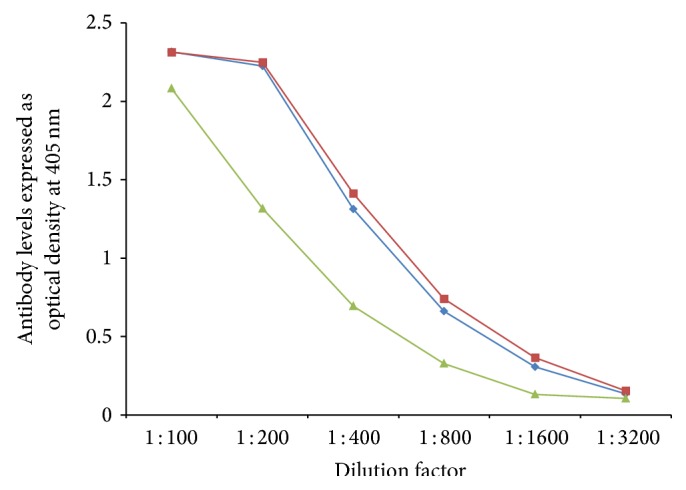
Serial dilution of IgG (blue diamond), IgA (green triangle), and IgM (red square) antibody against soy.

**Figure 8 fig8:**
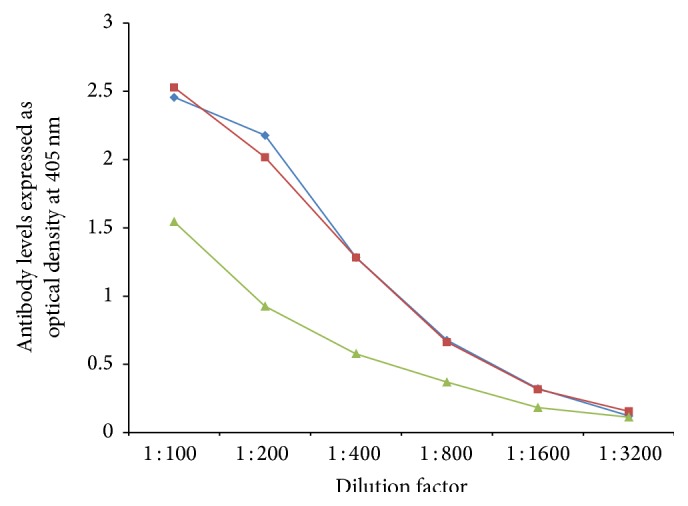
Serial dilution of IgG (blue diamond), IgA (green triangle), and IgM (red square) antibody against corn.

**Figure 9:**
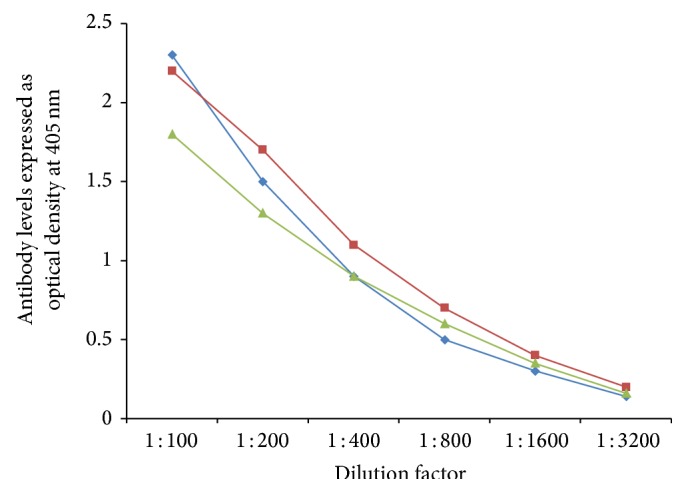
Serial dilution of IgG (blue diamond), IgA (green triangle), and IgM (red square) antibody against spinach.

In order to demonstrate specificity of detected antibody and to rule out nonspecific reaction, in addition to neural cell antigens and aquaporins, all sera were reacted with wells coated with HSA and OVA peptide 323–339, followed by the addition of all reagents in the ELISA. ODs for all tested sera, after reaction with HSA or OVA peptide, were less than 0.2. Additionally, serial dilution of 1 : 100–1 : 3200 of sera with high levels of antibodies against each aquaporin was performed. Results depicted in Figures 5 [Fig fig6] [Fig fig7] [Fig fig8]–9 showed that, in proportion to dilution, a significant decline in antibody reactivity was observed.

**Figure 10:**
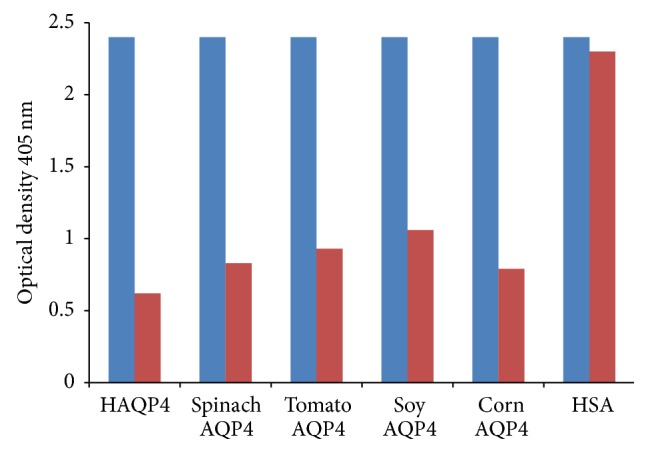
Inhibition of human AQP4 IgG antibody with human AQP4, spinach AQP4, tomato AQP4, soy AQP4, corn AQP4, and HSA. Controls = blue column; patients = red column.

In addition, inhibition by specific and nonspecific antigen was conducted by the addition of either HSA, human AQP4, or each plant aquaporin to three different sera with a very high level of IgG antibody against human AQP4. The data summarized in Figure 10 show that while HSA did not cause any inhibition of human anti-AQP4 binding to ELISA wells coated with human AQP4, the addition of human AQP4 and corn, spinach, tomato, and soy aquaporins to the same sera resulted in inhibition of antibody-antigen reaction by 75%, 67%, 65%, 61%, and 56%, respectively (Figure 10).

## 4. Discussion

In an earlier study [28], it was shown that several proteins in nature have a significant similarity in sequence and structure to human AQP4. The researchers found that IgG from the sera of patients with NMO cross-reacted with a sequence found in plant aquaporins and that this reactivity was much higher in NMO patients than in controls. However, only 9 patients and 9 controls were involved in this study [28], and no IgM or IgA antibody measurements were done. For this reason, we wanted to examine whether or not this immunoreactivity to aquaporins is unique to NMO or could also be detected in patients with RRMS. Therefore, we studied IgG, IgM, and IgA isotype antibodies in the sera of 47 patients with RRMS against human AQP4, against plant AQP4 from soy, corn, spinach, and tomato, and against neural antigens such as MBP, MOG, and S100B.

Elevation in antibodies against MBP, MOG, and alpha-B-crystallin have been shown as an aid in the diagnosis and prognosis of MS [30, 31].

S100B and AQP4 are both astrocytic proteins that enter the bloodstream when there is a disruption of the BBB. This entry of S100B and AQP4 into the bloodstream can result in the production of antibodies against them [35–38].

Based on these studies, we tested the presence of antibodies against human and plant aquaporins in RRMS patients and examined their correlation with other brain-specific antibodies detected in a subgroup of MS. As shown in Figures 1–3 and Table 1, a significant percentage of RRMS patients showed elevation not only in antibodies against human AQP4 and the aquaporins of soy, corn, tomato, and spinach, but also against MBP, MOG, and S100B. At this point, it is not known whether the antibodies are reacting first to human AQP4 and then cross-reacting with plant AQP4 or vice versa. There is always a possibility that this reaction against the specific AQP4 peptides used in this study is an epiphenomenon. We think, however, that this probability is slight, not only because we detected elevations against human and plant aquaporins and also against MBP, MOG, and S100B, but because we also detected very high correlations between the aquaporins and the neural antigens.

To test this association between the elevations of antibodies against MBP, MOG, S100B, human AQP4, and plant aquaporins, we used a simple linear regression model between each such pair and calculated *R* ^2^ values and *p* values (Tables 2–4). Data presented in these tables show *R* ^2^ of 0.586 to 0.831 for IgG, 0.513 to 0.941 for IgA, and 0.778 to 0.947 for IgM. This regression analysis suggests a relationship between antibodies against the food and brain proteins and peptides with the highest correlation between S100B and soy AQP4. The relationship between each antibody is also shown in a 2-way cluster analysis of Pearson's correlation coefficients between the food and brain proteins. Each isotype antibody is clustered together with high correlations between the food and brain antibodies, with the IgM antibody reactivity between the 2 groups being the most significant (Figure 4).

To support the importance of these AQP4 antigenic epitopes in immunoreactivity, we used 3 different commercially available monoclonal antibodies made against human AQP4 aa 1–22, AQP4 aa 200–300, and AQP4 aa 249–323, and reacted them with human and plant AQP4 peptides described in the Vaishnav study [28]. Only antibody made against peptide aa 200–300 reacted strongly against both human and all 4 plant aquaporins. Antibody against peptide aa 249–323 resulted in weak reactivity against human, corn, and soy aquaporins, while antibody made against peptide aa 1–22 did not react at all with any of the AQP4 peptides used in our study. This shows heterogeneity in antibody reaction against various AQP4 epitopes.

Another study by Iorio et al. [39] found that antibody against AQP4 extracellular loop peptide aa 137–157 was also restricted to patients with NMO. This peptide was not used in either the Vaishnav et al. study [28] or this present study. Using antibody bound to live M1 and M23 cells, Iorio's group found that while NMO serum bound to 100% of the AQP4 in live cell membranes, only 47% of the NMO sera reacted with peptides originating from loops A, C, and E using ELISA and Western Blot, with the detected loop C antibody being highly specific to NMO.

Therefore, for the differentiation of NMO from classic MS, RRMS, and other neuroimmune disorders, it is crucial to use live cell or tissue-based assays employing native AQP4, rather than assays utilizing peptides selected from extracellular or intracellular loops. However, these assays could not be used for cross-reactivity studies between plant proteins and human tissue antigens and as aids in possible dietary manipulation in autoimmune disorder treatment protocols.

In sum, while cell-based assays for AQP4 IgG antibodies are more specific to NMO, our present study shows that IgG, IgA, and, in particular, IgM antibodies against AQP4 peptides can also be detected in patients with MS. It is possible that exposure to epitopes that resemble human AQP4 from exogenous sources such as plants may play a role in the etiology of RRMS and possibly other autoimmune disorders. Although an association between plant antigens and autoimmune diseases has been previously suggested for celiac disease, lupus, scleroderma, type I diabetes, and MS [40–44], there is need for further evaluation of the role of plant proteins in the generation of cross-reactive antibodies against human AQP4, S100B, MOG, and MBP and the consequent development of RRMS and other neuroimmune disorders. This may help in the development of dietary guidelines for dietary modifications for patients with neuroimmune disorders.

## Figures and Tables

**Table 1:** Measurement of antibodies against MBP, MOG, S100B, human AQP4, soy AQP4, corn AQP4, tomato AQP4, and spinach AQP4 in controls (C) and patients (P) with RRMS expressed by ELISA optical densities and mean ± SD.

		MBP (OD)		MOG (OD)		S100B (OD)		Human AQP4 (OD)		Soy AQP4 (OD)		Corn AQP4 (OD)		Tomato AQP4 (OD)		Spinach AQP4 (OD)	
		C	P	C	P	C	P	C	P	C	P	C	P	C	P	C	P
IgG	Low	0.19	0.31	0.06	0.31	0.20	0.31	0.16	0.31	0.05	0.24	0.17	0.15	0.05	0.23	0.17	0.26
	High	1.43	2.20	1.15	2.18	1.33	2.44	1.97	2.28	1.13	2.84	1.31	2.26	1.13	2.73	1.45	2.44
	Mean ±	0.75	1.07	0.55	0.91	0.67	1.04	0.81	1.02	0.54	1.07	0.69	1.08	0.52	1.02	0.76	1.15
		±0.25	±0.49	±0.23	±0.45	±0.25	±0.51	±0.36	±0.5	±0.24	±0.57	±0.26	±0.53	±0.26	±0.56	±0.28	±0.48
	*p* value	<0.0001		<0.0001		<0.0001		0.0093		<0.0001		<0.0001		<0.0001		<0.0001	

IgA	Low	0.13	0.12	0.12	0.10	0.14	0.11	0.21	0.29	0.13	0.13	0.22	0.26	0.13	0.15	0.06	0.27
	High	1.05	3.72	0.93	2.98	1.18	2.99	2.01	3.35	1.01	3.36	1.33	2.98	1.03	2.56	1.73	3.04
	Mean ±	0.61	0.94	0.55	0.87	0.57	0.85	0.67	1.11	0.49	0.76	0.66	0.96	0.50	0.81	0.62	0.96
		±0.23	±0.71	±0.19	±0.51	±0.23	±0.58	±0.34	±0.68	±0.20	±0.54	±0.30	±0.57	±0.20	±0.52	±0.31	±0.58
	*p* value	0.0015		<0.0001		0.0015		<0.0001		0.0008		0.0009		0.0001		0.0003	

IgM	Low	0.16	0.48	0.06	0.55	0.05	0.38	0.17	0.42	0.06	0.35	0.14	0.35	0.05	0.36	0.16	0.61
	High	1.13	2.88	1.37	3.40	1.25	3.52	1.40	2.76	1.18	3.53	1.80	3.39	1.16	2.90	1.49	3.58
	Mean ±	0.63	1.16	0.64	1.41	0.62	1.19	0.76	1.18	0.53	1.07	0.71	1.34	0.61	1.15	0.82	1.52
		±0.25	±0.61	±0.28	±0.79	±0.25	±0.74	±0.29	±0.57	±0.22	±0.70	±0.32	±0.76	±0.23	±0.60	±0.33	±0.76
	*p* value	<0.0001		<0.0001		<0.0001		<0.0001		<0.0001		<0.0001		<0.0001		<0.0001	

**Table 2:** Results of the simple linear regression between each pair of lgG isotypes of the food proteins and brain proteins in RRMS patients. The first number in each cell presents corresponding Pearson's correlation coefficient and the second number in parentheses presents its *p* value. Small *p* values (less than 0.05) are marked in bold. Note that *R* ^2^ values of these regressions are the squares of Pearson's correlation coefficients.

IgG	MBP (OD)	MOG (OD)	S100B (OD)	Human AQP4 (OD)
Soy AQP4	0.6362 **(<0.0001)**	0.7897 **(<0.0001)**	0.8312 **(<0.0001)**	0.6031 **(<0.0001)**
Corn AQP4	0.6033 **(<0.0001)**	0.7912 **(<0.0001)**	0.8038 **(<0.0001)**	0.6323 **(<0.0001)**
Tomato AQP4	0.8040 **(<0.0001)**	0.6361 **(<0.0001)**	0.6479 **(<0.0001)**	0.6163 **(<0.0001)**
Spinach AQP4	0.5939 **(<0.0001)**	0.7732 **(<0.0001)**	0.8232 **(<0.0001)**	0.5860 **(<0.0001)**

**Table 3:** Results of the simple linear regression between each pair of lgA isotypes of the food proteins and brain proteins in RRMS patients. The first number in each cell presents corresponding Pearson's correlation coefficient and the second number in parentheses presents its *p* value. Small *p* values (less than 0.05) are marked in bold. Note that *R* ^2^ values of these regressions are the squares of Pearson's correlation coefficients.

IgA	MBP (OD)	MOG (OD)	S100B (OD)	Human AQP4 (OD)
Soy AQP4	0.6651 **(<0.0001)**	0.9281 **(<0.0001)**	0.9419 **(<0.0001)**	0.7421 **(<0.0001)**
Corn AQP4	0.7035 **(<0.0001)**	0.9317 **(<0.0001)**	0.8868 **(<0.0001)**	0.6903 **(<0.0001)**
Tomato AQP4	0.6727 **(<0.0001)**	0.8862 **(<0.0001)**	0.8700 **(<0.0001)**	0.7692 **(<0.0001)**
Spinach AQP4	0.5136 **(<0.0001)**	0.6677 **(<0.0001)**	0.6758 **(<0.0001)**	0.6608 **(<0.0001)**

**Table 4:** Results of the simple linear regression between each pair of lgM isotypes of the food proteins and brain proteins in MS patients. The first number in each cell presents corresponding Pearson's correlation coefficient and the second number in parentheses presents its *p* value. Small *p* values (less than 0.05) are marked in bold. Note that *R* ^2^ values of these regressions are the squares of Pearson's correlation coefficients.

IgM	MBP (OD)	MOG (OD)	S100B (OD)	Human AQP4 (OD)
Soy AQP4	0.9502 **(<0.0001)**	0.9290 **(<0.0001)**	0.9475 **(<0.0001)**	0.8887 **(<0.0001)**
Corn AQP4	0.7823 **(<0.0001)**	0.8582 **(<0.0001)**	0.7788 **(<0.0001)**	0.8346 **(<0.0001)**
Tomato AQP4	0.8771 **(<0.0001)**	0.9297 **(<0.0001)**	0.8668 **(<0.0001)**	0.9089 **(<0.0001)**
Spinach AQP4	0.8549 **(<0.0001)**	0.9184 **(<0.0001)**	0.8567 **(<0.0001)**	0.8732 **(<0.0001)**
